# Preparation of Polyethylene Clay Composites via Melt Intercalation Using Hydrophobic and Superhydrophobic Organoclays and Comparison of Their Textural, Mechanical and Thermal Properties

**DOI:** 10.3390/polym16020272

**Published:** 2024-01-19

**Authors:** Ahmet Gürses, Kübra Güneş

**Affiliations:** Department of Chemistry Education, K, K Education Faculty, Atatürk University, Erzurum 25240, Turkey; kgunes@atauni.edu.tr

**Keywords:** nanocomposite, thermal and mechanical properties, polymer clay nanocomposites, cetyltrimethylammonium bromide, organoclay, hydrophobicity, layered clays

## Abstract

Polymer clay nanocomposites, which can exhibit many superior properties compared to virgin polymers, have gained increasing interest and importance in recent years. This study aimed to prepare composites of two organoclays with unusual ratios and different degrees of lyophilicity with low-density polyethylene and compare their textural structures and thermal and mechanical properties with those of virgin polymer. For this purpose, firstly, organoclays, hydrophobic and superhydrophobic organoclays (OC and SOC), were prepared by solution intercalation method using cetyltrimethylammonium bromide with and without addition of a hydrocarbon substance. Then, using both organoclays, polyethylene organoclay composites were prepared and characterized using X-ray powder diffraction (XRD), high-resolution transmission electron microscopy (HRTEM) and Fourier transform infrared spectroscopy (FTIR) techniques. Additionally, tensile and hardness tests were performed to determine the mechanical properties of the composites, and differential scanning calorimetry (DSC) thermograms were taken to examine their thermal behavior. XRD patterns and HRTEM images of hydrophobic and superhydrophobic organoclays and the composites show that the characteristic smectite peak of the clay shifts to the left and expands, that is, the interlayer space widens and, in the composites, it deforms immediately at low clay ratios. HRTEM images of the composites prepared especially with low clay ratios indicate that a heterogeneous dispersion of clay platelets occurs, indicating that nanocomposite formation has been achieved. On the contrary, in the composites prepared with high clay ratios, this dispersion behavior partially turns into aggregation. In the composites prepared using up to 20% by weight of superhydrophobic organoclay, extremely stable and continuous improvements in all mechanical properties were observed compared to those of the composites prepared using hydrophobic organoclay. This indicates that by using superhydrophobic organoclay, a ductile nanocomposite of polyethylene containing inorganic components in much higher than usual proportions can be prepared.

## 1. Introduction

Polymer and polymer mixtures, which are interesting and widely used materials, have many superior properties compared to other materials, and polymer mixtures can also show the properties of single-phase materials and are defined as homogeneous alloys at the molecular level [1,2]. High-performance polymer nanocomposites have the fastest demand among engineering products and an estimated annual growth rate of 25%. The main application areas of polymer nanocomposites, which have a very high potential in many areas from packaging to biomedical applications, are the automotive industry, packaging, aviation and space applications and the construction industry. There is also a great increase in their use in various sectors such as medicine, sensing, smart structures and textiles, military weapons, energy production and management and aerodynamics [3,4]. However, broader applications of these materials are limited by technological challenges related to low-cost improvement prospects [5]. Polymer clay nanocomposites (PNCs), which are among the most promising materials for a number of applications, have attracted the attention of scientists in recent years due to their superior thermal, mechanical, magnetic, optical, dielectric or electromagnetic and barrier properties compared to virgin polymers and traditional microcomposites [6,7,8,9,10]. Today, polymer clay nanocomposites are considered priority systems because clay has a high aspect ratio and a flat morphology [11]. In general, polymer nanocomposite product transformations can be beneficial in improving the mechanical properties of polymers as well as physical properties such as thermal stability, fire retardant, gas barrier and corrosion protection [12,13,14,15,16].

Montmorillonite is a smectite group clay with a 2:1 layered structure consisting of Mg^2+^ or Al^3+^ coordinated with oxygen atoms, with an octahedral layer between them and coordination bonds between silicon (Si4+) and oxygen atoms [17,18]. Si-OH and Al-OH groups have a primary role in the preparation of organoclays, even if the methods are different [17,18,19]. Bentonite-type layered clays, especially montmorillonite and illite, which have high ion exchange capacity and high specific surface area, are widely used in the preparation of polymer clay nanocomposites as well as in different applications such as cement, ceramic products, paint and paper production [20,21,22,23,24,25,26,27]. However, the hydrophilic nature of clay hinders the effective dispersion of clay platelets in most thermoplastics. Therefore, surface modification is needed to reduce the clay–polymer interface energy and make it organophilic [28]. The most common surfactants used to prepare organoclay are cationic (especially quaternary ammonium salts), zwitterionic and nonionic. When using cationic and zwitterionic surfactants, clays are modified through the exchange of exchangeable cations present in the interlayer space, whereas in the case of nonionics, modification can occur via dipole–ion interactions, thus leading to hybrid materials with dual behavior, hydrophilic-hydrophobic [29,30,31,32,33].

Polymer nanocomposites (PNCs) have shown an overwhelming rise in their usage in various military and defense sectors such as military medicine, sensing, smart structures and textiles, military weapons, power generation and management and aerodynamics [4] The broader applications of these materials are limited due to the technological difficulties that are related to the low-cost improvement expectations [5]. Polymer/clay nanocomposites (PNCs) constitute a new class of composite materials that have been studied in recent years due to their superior thermal, mechanical and barrier properties compared to virgin polymer or traditional microcomposites [6,7,8,9]. Currently, the PNC material is considered to be an encouraging system since the clay has a high aspect ratio and a platy morphology [11]. PNCs can be useful for boosting the physical properties that belong to polymers, e.g., thermal stability [12], fire retardant [13], gas barrier [14] and corrosion protection [12] as well to improve the mechanical properties of polymers [16]. Bentonites that consist mainly of montmorillonite and illite are the accepted fillers in polymer/clay nanocomposites and have different applications such as cements, ceramic products, paints and paper [20,21,22,23,24,25]. Bentonites are widely used clays due to their large ion exchange capacity and high specific surface area [26,27]. However, their hydrophilic nature acts as a barrier to the effective dispersion of clay platelets in most thermoplastics. The surface modification that is necessary to decrease the interfacial energy makes them organophilic by decreasing the wettability of clays [28]. The quaternary alkyl ammonium salts that are the most used cationic surfactants to prepare organoclays are frequently used to replace the exchangeable ions in the interlayer spaces of clay [31,32,33].

Although various polymers are used as a polymeric matrix in the preparation of PNCs, polyethylene, which has good mechanical properties, a light weight, easy processability, a low cost, high chemical resistance and abrasion resistance, is one of the most widely used thermoplastics [34]. However, their non-polar skeletal structure and lyophilicity may require further lyophilization of organically modified silicates or clays for the preparation of nanocomposites [35,36]. One of the most suitable techniques used in the preparation of the composite of modified bentonite and thermoplastic polyethylene is melt intercalation [37]. Melt intercalation involves the annealing of a mixture of polymer and organically modified material, either statically or under shear, above the softening point of the polymer [38]. It is environmentally friendly as it requires no solvents and is a low-cost process due to its compatibility with existing processes. Preparation of nanocomposites requires extensive delamination of the layered clay structure and good and uniform dispersion of the resulting platelets within the polymer matrix. Preparation of nanocomposites by conventional polymer processing processes therefore requires a strong interfacial interaction between the polymer matrix and clay to generate shear forces of sufficient strength [39].

Although many nanocomposites consisting of lyophilic polymer polyethylene and clay have been prepared, those prepared with especially high amounts of super lyophilized organoclay have not been encountered very often Therefore, the presented work focused on the preparation of polyethylene clay composites via melt intercalation using hydrophobic and superhydrophobic organoclays and their textural, structural, mechanical and thermal characterization, as well as examined the effect of organoclay content, especially on mechanical properties.

## 2. Materials and Methods

### 2.1. Materials

To prepare hydrophobic and superhydrophobic organoclays (OC and SOC) and polymer composites, cationic surfactant, cetyltrimethylammonium bromide (CTAB) (Merc. Co., Darmstadt, Germany), high-density polyethylene (LDPE) (Merc Co.), raw clay and a hydrocarbon material were used. Raw clay, which consists of smectite (26%), chlorite (20%), illite (17%), kaolinite (14%), analcime (11%), calcite (7%), feldspar (3%) and quartz (3%), was obtained from the Oltu region of Erzurum city in Turkey. For the XRF analysis of clay, a sequential Thermo ARL Performx model WD-XRF spectrometer, Waltham, MA, USA (Rh anticathode X-ray tube (4.2 kW), maximum voltage 60 kV, maximum current 120 mA and a typical measuring time of 0.1–165 s per element and total angle range of 0–154°) was used. The X-ray fluorescence (XRF) composition of the clay is also given in Table 1.

The cation exchange capacity (CEC) of raw clay was determined using the methylene blue test (ANSI/ASTM C837-76) [40] and is given in Table 2 along with some other physical properties. Additionally, some properties of the hydrocarbon material used in the preparation of superhydrophobic organoclay are given in Table 3.

### 2.2. Method

#### Preparation of Hydrophobic and Superhydrophobic Organoclay

The raw clay, purified by the washing method [41], was dried in a vacuum oven and sieved through ASTM sieves to obtain a 38–85 µm size fraction. To prepare hydrophobic clay, 50 g of raw clay was added into 50 L of CTAB aqueous solution (160 mg/L). The mixture was then filtered, dried in a vacuum oven at 100 °C for 2 h, ground and passed through a 400-mesh sieve. While preparing superhydrophobic organoclay, a stable suspension was obtained by adding 12.5 g of long-chain hydrocarbon in the mixing stage before the addition of raw clay, and then the same processes were repeated by adding clay. Static contact angle measurements of organoclays (OC and SOC) were performed using an optical goniometer (CAM-101, KSV Instruments, Helsinki, Finland). For this, 0.4 g powder samples were pelleted under 1.06 ton/cm^2^ pressure and the contact angle was calculated using the Young–Laplace equation by taking the image of a 6-µL water droplet falling on the solid. Static contact angles of the raw clay and the prepared hydrophobic and superhydrophobic organoclays were measured as <30°, >105° and >115°, respectively.

### 2.3. Preparation of Polyethylene/Organoclay Composites

#### Melt Intercalation

**Figure 1:**
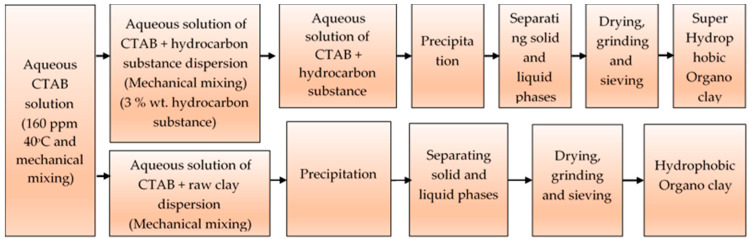
Schematic representation of hydrophobic organoclay and superhydrophobic organoclay preparation procedures.

A certain amount of hydrophobic and superhydrophobic organoclays (OC and SOC) were blended with high-density granulated polyethylene (LDPE) in separate containers with the addition of a very small amount of glycerin, and then poured into the bunker of the single screw extruder, heated to 190–210 °C, and the melt intercalation process was performed at a screw speed of 150 min^−1^. The hot melts coming out of the extruder were mechanically pressed. Schematic representation of the hydrophobic organoclay and superhydrophobic organoclay preparation procedures is shown in Figure 1.

The loading ratios of organoclays were adjusted as 5.0, 10.0, 15.0, 20.0 and 25.0 wt.%, based on the total mixture. The codes and contents of organoclay and low-density polyethylene composite samples are given in Table 4.

### 2.4. Characterization of Polyethylene Clay Composites

HRTEM, FTIR, XRD and DSC techniques were used for textural, structural, crystallographic and thermal characterization of the raw clay, the organoclays and the composites.

HRTEM images were taken using a JEOL 2100 high-resolution transmission electron microscope, Boston, MA, USA (HRTEM) (LaB6filament) at 200 Kv.

The XRD patterns were measured in the range of 2θ 2–40° and at the scanning speed of 4/min, using a Rigaku 2200D/max (Rigaku Co., Tokyo, Japan) powder diffractometer with Cu Kα (1.540 Å) radiation operating at 5 kV and 40 mA.

To obtain FTIR spectra, the Perkin–Elmer Spectrum-One spectrometer (Waltham, MA, USA) and KBr palletization method were used, with an average of 100 scans for the range of 400–4000 cm^−1^ and a resolution of 1 cm^−1^ at a scan rate of 20 min^−1^.

DSC thermograms were taken using a differential scanning calorimeter (DSC 7020, Hitachi, Ibaraki, Japan) with a typical sample weight of approximately 10 mg and a scan rate of 20 °C min^−1^.

Hardness values were measured using Shore hardness, a measure that determines the resistance to penetration by a needle under a spring force. Flexible and rigid types are indicated by the letter A and the letter D, with a scale defined as a number from 0 to 100 on the A or D scale. Measurements were made using the Shore D hardness scale at an ambient temperature. Also, scratch tests were carried out by rubbing the Rockwell C diamond tip against the coatings at a speed of 0.007 m/s under a constant normal load of 5 N.

Tensile test, modulus of elasticity, tensile strength, yield strength and elongation percentage were determined by applying a 5-kN load at ambient temperature, at a speed of 2 mm min^−1^, with dog bone–type molds using a Shimadzu AG-100kNIS test instrument (Kyoto, Japan) (ASTM 638 M-91a [42]).

## 3. Results and Discussion

### 3.1. HRTEM Analysis

**Figure 2:**
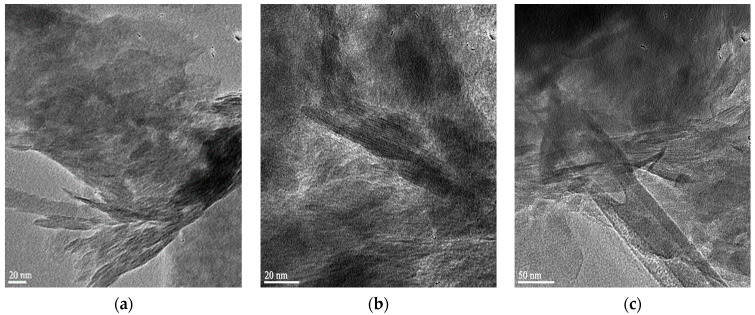

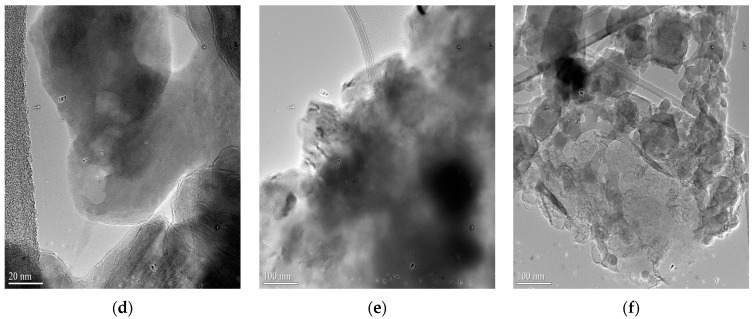
HRTEM images of (**a**) raw clay (RC), (**b**) hydrophobic clay (OC), (**c**) superhydrophobic clay (SOC), (**d**) virgin polymer (LDPE), (**e**) the composite prepared using hydrophobic organoclay (LDPEOCC1) and (**f**) the composite prepared using superhydrophobic organoclay (LDPESOCC1).

HRTEM images of raw clay (RC), both organoclays (OC and SOC), virgin polymer (LDPE) and the composites (LDPEOCC1 and LDPESOCC1) prepared using different ratios of hydrophobic and superhydrophobic organoclays are shown in Figure 2, respectively.

The dark fibrous structures in the images in Figure 2a–c represent the layers that make up the clay grains [43]. From the HRTEM images of raw clay, hydrophobic organoclay and superhydrophobic organoclay, it was seen that the increase in interlayer distance was greater in superhydrophobic organoclay compared to hydrophobic organoclay. In addition, it was observed that the clay layers are more tightly stacked in raw clay; in hydrophobic organoclay, partially spaced regular layer stacks are formed; and in superhydrophobic organoclay, the layers are more irregular and more spaced.

Comparing the HRTEM images of the virgin polymer and the composites prepared with 5 wt.% hydrophobic organoclay and superhydrophobic organoclay (Figure 2d–f), it can be seen that the textural structures of the virgin polymer (LDPE) and the composite containing hydrophobic organoclay (LDPEOCC1) are relatively similar. This comparison also reveals that while the platelet dispersion in the composite containing hydrophobic organoclay is tactoidal and more localized in stacks, the dispersion in the composite containing superhydrophobic organoclay is more homogeneous and more separated.

### 3.2. FTIR Analysis

**Figure 3:**
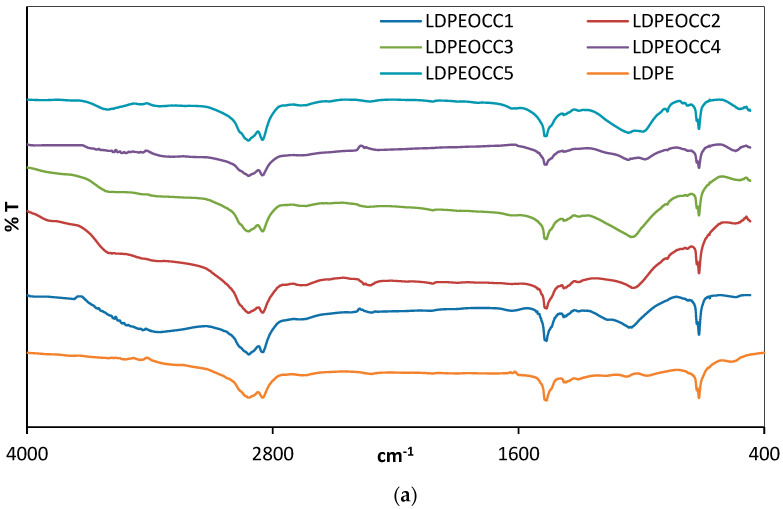

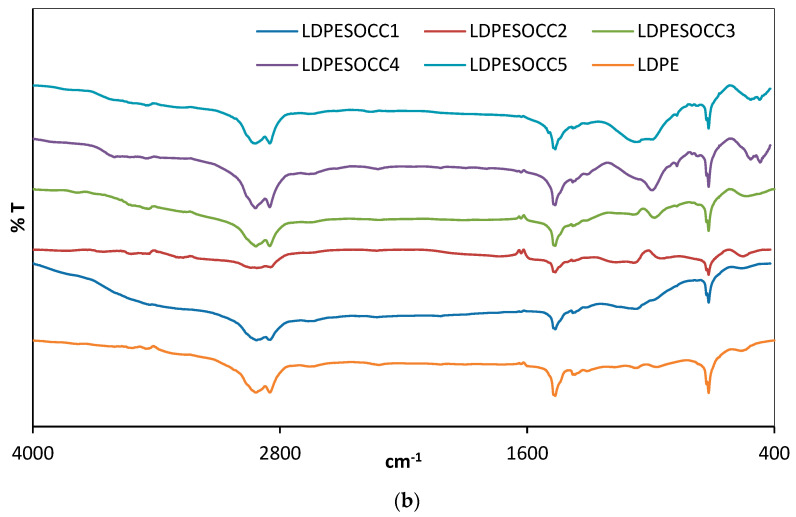
FTIR spectra of the virgin polymer (LDPE) and the composites prepared using both hydrophobic organoclay and superhydrophobic organoclay (LDPEOCC1–5 (**a**) and LDPESOCC1–5 (**b**)).

FTIR spectra of the virgin polymer (LDPE) and the composites (LDPEOCC1–5 and LDPESOCC1–5) prepared using various ratios of hydrophobic and superhydrophobic organoclays are given in Figure 3a,b.

Unlike the spectrum of the virgin polymer, in the FTIR spectra of the composites prepared with various ratios of hydrophobic and superhydrophobic organoclays (5, 10, 15, 20 and 25% by weight), in addition to the four main peaks, three new peaks appeared at 2913, 2916 and 1463 cm^−1^.

In the spectra of the polyethylene hydrophobic and superhydrophobic organoclay composites (LDPEOCC1–5 and LDPESOCC1–5), the peaks at 2913 and 2916 cm^−1^ are related to CH_2_ asymmetric and symmetric vibrations, and the peak at 1464 cm^−1^ is related to H-C-H stretching vibration [44]. The peaks at 3649 and 3489 cm-^1^ can be attributed to free and hydroxyl stretching vibrations, and the peak at 1643 cm^−1^ to the bending vibrations of adsorbed water molecules (H–O–H). Additionally, the peaks at 1030 and 1045 cm^−1^ are related to Si-O stretching and the peak at 718 cm^−1^ is related to Al-Al-OH bending vibrations at the edges of the clay layers. Moreover, the peaks at 532 and 444 cm^−1^ can also be associated with Si–O–Al and Si–O–Si bending vibrations. These peaks broaden and increase in intensity with increasing clay content in both composites.

### 3.3. XRD Analysis

X-ray Diffraction (XRD) analysis is widely used to determine the change in the matrix and the interlayer distance by comparing the diffraction peaks of crystalline structures [45].

**Figure 4:**
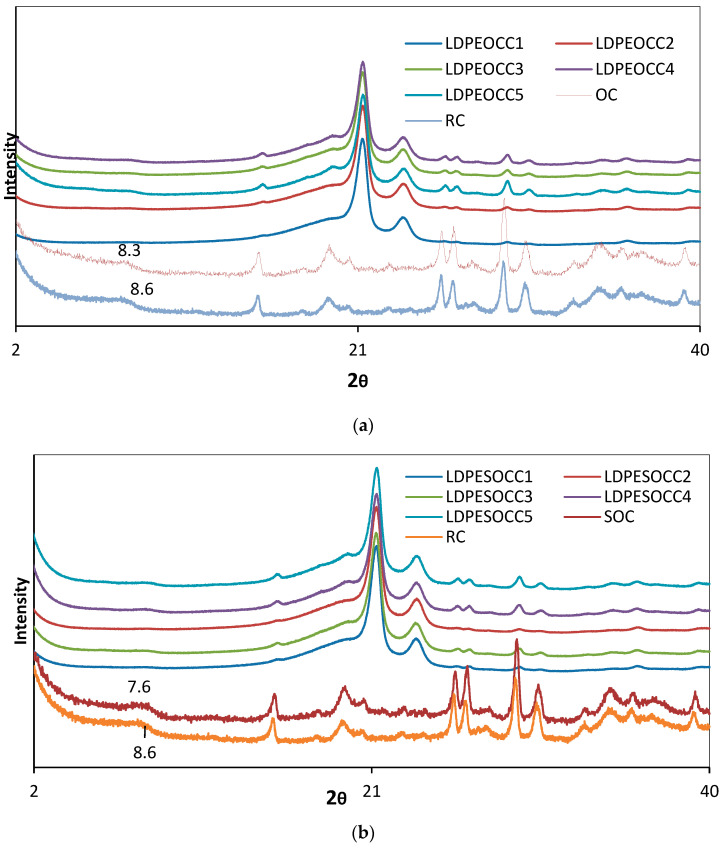
XRD patterns of raw clay (RC) and the composites prepared using both hydrophobic organoclay and superhydrophobic organoclay: (**a**) LDPEOCC1–5 and (**b**) LDPESOCC1–5.

Figure 4 shows the XRD patterns of raw clay and the composites (a and b) prepared using various ratios of hydrophobic organoclay and superhydrophobic organoclay. It can be seen from Figure 4a,b that the XRD patterns of both hydrophobic and superhydrophobic organoclays and the XRD patterns of the composites prepared using both organoclays are quite similar to each other.

From this figure, it can be seen that the characteristic smectite peak at 8.6°, which was chosen to monitor the crystallographic changes of raw clay and organoclays, shifted to 8.3° and 7.6° for hydrophobic and superhydrophobic clays, respectively, with the lyophilization process. This indicates that the interlayer distance widens. From the XRD patterns of the composites (a and b) prepared using hydrophobic organoclay and superhydrophobic organoclay, it can be seen that at low organoclay ratios, the smectite peak almost completely disappears (up to 15%).

However, it has been observed that as the organoclay ratio increases, the peak reappears, due to the polymer chains not being intercalated effectively in the interlayer spaces of the clay, especially at the ratios of 20 and 25%.

### 3.4. DSC Analysis

Differential scanning calorimetric measurements are widely used to examine various changes such as glass transition or softening, melting, crystallization, curing and degradation during heating of organoclay carbon nanotubes and polymer nanocomposites [46].

**Figure 5:**
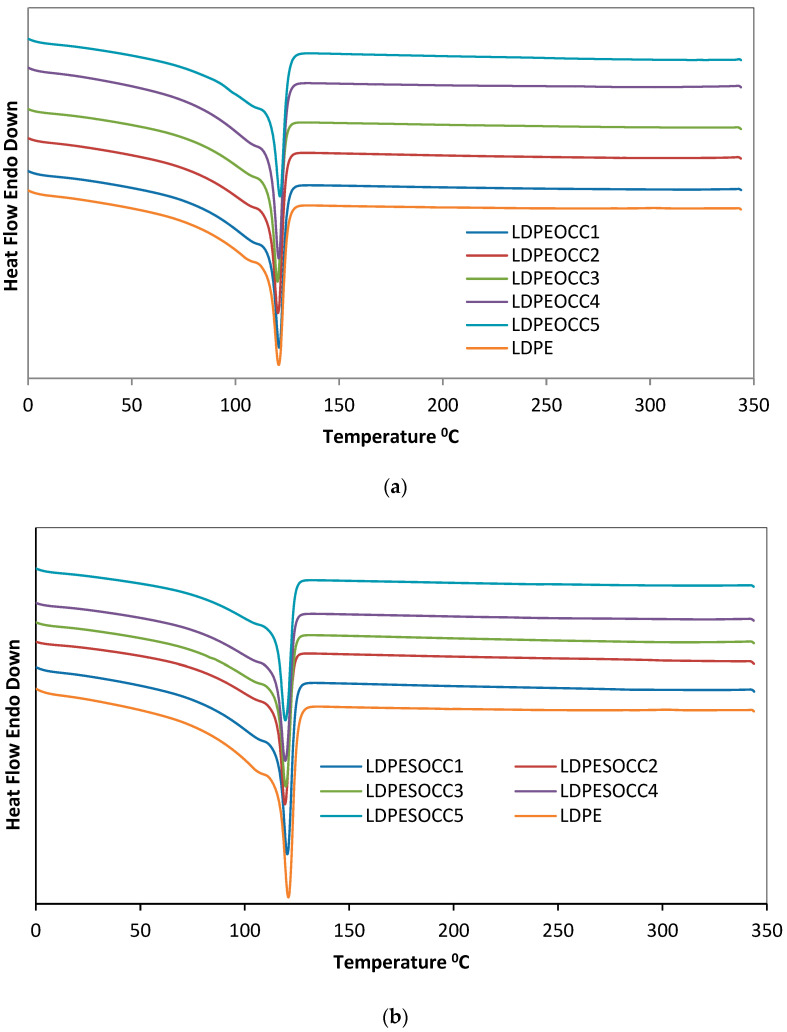
DSC thermograms of the virgin polymer (LDPE) and the composites prepared using both hydrophobic organoclay and superhydrophobic organoclay: (**a**) LDPEOCC1–5 and (**b**) LDPESOCC1–5.

Thermograms of the virgin polymer (LDPE) and the polymer organoclay composites (LDPEOCC1–5 and LDPESOCC1–5) prepared using hydrophobic and superhydrophobic organoclays are given in Figure 5a,b, respectively.

From Figure 5a,b, it can be seen that there is no significant change in the thermal behavior of the composites prepared using both organoclays (LDPEOCC1–5 and LDPESOCC1–5) compared to the virgin polymer (LDPE), and that no significant change in the crystalline behavior of the polymer occurs even at high organoclay content.

However, compared to the virgin polymer, in both composite samples, the transition endotherms in the melting zone did not change significantly, but the glass transition temperatures (T_g_) shifted slightly towards higher temperatures with the increase in the organoclay ratio. This can be attributed to the homogeneous dispersion of clay platelets acting as a shield against heat conduction into the crystallites. It can also be suggested that exfoliated or tactoidally dispersed organoclay platelets inhibit the growth of polymer crystal structures [47,48].

### 3.5. Mechanical Analysis

The mechanical properties of composites largely depend on the degree of dispersion of the clay plates and the effectiveness of adhesion between the clay surface and polymer chains [49,50]. Table 5 shows the findings from mechanical tests such as tensile strength, yield strength, modulus of elasticity, % elongation, scratching and Shore hardness conducted on the virgin polymer (LDPE) and the composites (LDPEOCC1–5 and LDPESOCC1–5) prepared using hydrophobic and superhydrophobic organoclays. The effect of the organoclay ratio significantly depends on the degree of polymer–clay intercalation [51]. The composites containing 5% by weight hydrophobic organoclay showed significant improvements in all mechanical properties compared to the virgin polymer. However, as the hydrophobic organoclay ratio increased, a serious decrease in mechanical values was observed. This can be predominantly attributed to the possible aggregation of clay layers and resulting phase separations at unusually high hydrophobic organoclay content (higher than 10%).

On the other hand, the composites prepared using up to 20% by weight of superhydrophobic organoclay exhibit very stable and continuous improvements in all mechanical properties compared to the composites prepared using hydrophobic organoclay. In particular, the hardness values of the composites prepared at 10% and 15% superhydrophobic organoclay ratios by weight remained the same as the untreated polymer, and both the elastic modulus and % elongation values were found to be extremely high. This indicates that the interfacial energy between the polymer matrix and organoclay platelets decreases, resulting in excellent adhesion interactions. Moreover, these findings and evaluations are also consistent with the HRTEM images of the LDPEOCC1 and LDPESOCC1 nanocomposites in Figure 2 and the XRD patterns in Figure 4.

## 4. Conclusions

In this study, composites with an unusually high content of a lyophilic polymer such as polyethylene and organoclays with two different lyophilicity degrees were prepared and compared in terms of textural, structural, thermal and mechanical properties.

When comparing the HRTEM images of the virgin polymer and the composites prepared with 5 wt.% hydrophobic organoclay and superhydrophobic organoclay, it can be seen that the textural structures of the virgin polymer (LDPE) and the composite containing hydrophobic organoclay (LDPEOCC1) are relatively similar. On the other hand, while the platelet dispersion in the composite containing hydrophobic organoclay is tactoidal and more localized in stacks, the dispersion in the composite containing superhydrophobic organoclay is more homogeneous and more separated.

XRD patterns of the composites prepared using hydrophobic organoclay and superhydrophobic organoclay show that at low organoclay ratios, the characteristic smectite peak disappears almost completely (up to 15%). However, as the organoclay ratio increases, the peak reappears, due to the polymer chains not being intercalated effectively in the interlayer spaces of the clay, especially at the ratios of 20 and 25%.

The fact that there is no significant change in the thermal behavior of the composites prepared using both organoclays (LDPEOCC1–5 and LDPESOCC1–5) compared to the virgin polymer (LDPE) indicates that the extremely high organoclay content does not cause a significant change in the crystal structure of the polymer. However, compared to the virgin polymer, the transition endotherms in the melting zone did not change significantly in either type of composite, but the glass transition temperatures (T_g_) shifted slightly towards higher temperatures with increasing organoclay content. This is attributed to the homogeneous distribution of clay platelets acting as a shield against heat conduction to the crystallites.

The composites containing 5% by weight hydrophobic organoclay showed significant improvements in all mechanical properties compared to the virgin polymer. However, as the hydrophobic organoclay ratio increased, a serious decrease in mechanical values was observed. This has been predominantly attributed to the possible aggregation of clay layers and resulting phase separations at unusually high hydrophobic organoclay content (higher than 10%). In the composites prepared using up to 20% by weight of superhydrophobic organoclay, extremely stable and continuous improvements in all mechanical properties were observed compared to those of the composites prepared using hydrophobic organoclay. This means that by using superhydrophobic organoclay, a ductile nanocomposite of polyethylene containing much higher than usual proportions of inorganic components can be prepared.

## Figures and Tables

**Table 1:** The XRF composition of the raw clay.

Components (%)									
SiO_2_	Al_2_O_3_	CaO	MgO	Fe_2_O_3_	K_2_O	Na_2_O	TiO_2_	SO_3_	P_2_O_5_
56.77	15.16	8.44	8.79	0.48	4.04	4.20	0.84	0.91	0.37

**Table 2:** Some physical properties of the raw clay.

CEC ^a^	d ^b^	OMC ^c^	Liquid Limit,	Plastic Limit,	Plasticity	a ^d^
(meq/100 g)	(g/cm^3^)	(%)	w_L_ (%)	w_P_ (%)	index, I_p_	(m^2^/g)
48.90	2.61	5.10	102.00	35.00	67.00	64.20

^a^ Cation exchange capacity, ^b^ Specific gravity, ^c^ Organic matter content, ^d^ Specific surface area.

**Table 3:** Some characteristics of the long-chain hydrocarbon material.

Density	Calorific Value	Flash Point °C	Water by Distillation	C	H	N	S	Ash
(15 °C), kg/m^3^	MJ/kg		wt.%					
990.7	42.74	105.8	0.1	83.4	11.9	0.8	1.5	0.03

**Table 4:** Codes and contents of organoclay and low-density polyethylene composite samples.

Specimen Code	Nano Filler	(%wt.)
LDPE	-	-
(Low-Density Polyethylene)		
LDPEOCC1	Hydrophobic organoclay	5.0
(Hydrophobic organoclay and low-density polyethylene composite 1)		
LDPEOCC2	Hydrophobic organoclay	10.0
(Hydrophobic organoclay and low-density polyethylene composite 2)		
LDPEOCC3	Hydrophobic organoclay	15.0
(Hydrophobic organoclay and low-density polyethylene composite 3)		
LDPEOCC4	Hydrophobic organoclay	20.0
(Hydrophobic organoclay and low-density polyethylene composite 4)		
LDPEOCC5	Hydrophobic organoclay	25.0
(Hydrophobic organoclay and low-density polyethylene composite 5)		
LDPESOCC1		
(Superhydrophobic organoclay and low-density polyethylene composite 1)	Superhydrophobic organoclay	5.0
LDPESOCC2		
(Superhydrophobic organoclay and low-density polyethylene composite 2)	Superhydrophobic organoclay	10.0
LDPESOCC3		
(Superhydrophobic organoclay and low-density polyethylene composite 3)	Superhydrophobic organoclay	15.0
LDPESOCC4		
(Superhydrophobic organoclay and low-density polyethylene composite 4)	Superhydrophobic organoclay	20.0
LDPESOCC5		
(Superhydrophobic organoclay and low-density polyethylene composite 5)	Superhydrophobic organoclay	25.0

**Table 5:** Some mechanical properties of the virgin polymer (LDPE) and the composites prepared using both hydrophobic organoclay and superhydrophobic organoclay (LDPEOCC and LDPESOCC).

	Clay Ratio (%)	Hardness (Shore D)	Scratch Hardness (MPa)	Tensile Strength (MPa)	Yield Strength (MPa)	Elasticity Module (MPa)	Elongation (%)
LDPE	0	55.00	16,616	9.17	4.36	2.16	256.94
LDPEOCC	5	55.00	23,291	15.70	3.65	2.61	721.05
	10	56.33	15,000	12.41	5.28	2.61	336.66
	15	57.00	16,421	12.58	4.81	2.44	463.96
	20	56.40	18,051	7.14	3.74	1.83	141.05
	25	57.16	9527	7.83	3.81	0.69	232.51
LDPESOCC	5	56.16	14,317	11.52	3.45	1.70	510.39
	10	55.50	15,621	11.37	2.58	2.29	536.29
	15	55.83	16,041	12.96	4.83	1.64	582.92
	20	56.83	13,862	10.61	4.45	1.88	476.42
	25	56.33	7579	7.53	3.31	1.91	78.76

## Data Availability

The data presented in this study are available on request from the corresponding author.
